# Wide Linearity Range and Highly Sensitive MEMS-Based Micro-Fluxgate Sensor with Double-Layer Magnetic Core Made of Fe–Co–B Amorphous Alloy

**DOI:** 10.3390/mi8120352

**Published:** 2017-11-30

**Authors:** Lei Guo, Cai Wang, Saotao Zhi, Zhu Feng, Chong Lei, Yong Zhou

**Affiliations:** 1 Key Laboratory for Thin Film and Microfabrication of Ministry of Education, Department of Micro/Nano Electronics, School of Electronic Information and Electrical Engineering, Shanghai Jiao Tong University, Dongchuan Road 800, Shanghai 200240, China; g2252330@sjtu.edu.cn (L.G.); zhist1987@sjtu.edu.cn (S.Z.); feng.z@sjtu.edu.cn (Z.F.); 2 Material Science and Engineering School, Central South University, Changsha 410000, China; wangcai089@gmail.com

**Keywords:** magnetic sensor, micro-fluxgate sensor, MEMS, Fe–Co–B amorphous ribbon

## Abstract

This paper reports a novel micro-fluxgate sensor based on a double-layer magnetic core of a Fe–Co–B-based amorphous ribbon. The melt-spinning technique was carried out to obtain a Fe–Co–B-based amorphous ribbon composite of Fe_58.1_Co_24.9_B_16_Si_1_, and the obtained amorphous ribbon was then annealed at 595 K for 1 h to benefit soft magnetic properties. The prepared ribbon showed excellent soft magnetic behavior with a high saturated magnetic intensity (*B*_s_) of 1.74 T and a coercivity (*H*_c_) of less than 0.2 Oe. Afterward, a micro-fluxgate sensor based on the prepared amorphous ribbon was fabricated via microelectromechanical systems (MEMS) technology combined with chemical wet etching. The resulting sensor exhibited a sensitivity of 1985 V/T, a wide linearity range of ±1.05 mT, and a perming error below 0.4 μT under optimal operating conditions with an excitation current amplitude of 70 mA at 500 kHz frequency. The minimum magnetic field noise was about 36 pT/Hz^1/2^ at 1 Hz under the same excitation conditions; a superior resolution of 5 nT was also achieved in the fabricated sensor. To the best of our knowledge, a compact micro-fluxgate sensor with such a high-resolution capability has not been reported elsewhere. The microsensor presented here with such improved characteristics may considerably enhance the development of micro-fluxgate sensors.

## 1. Introduction

Magnetism detection and measurement have been an essential function in many application fields for years [1]. Among all the magnetic sensing methods, the application of the fluxgate principle constitutes one of the most important and well-developed detection techniques [2]. As a classical weak magnetic field measurement technology, fluxgate sensors have been attracting great interest worldwide because of their high sensitivity, high resolution, high temperature stability, low noise, and low offset drift [3]. However, traditional fluxgate sensors are fabricated by winding coils mechanically around magnetic cores, thus resulting in numerous drawbacks of the measuring systems based on conventional fluxgate sensors, such as large size, great weight, and high power consumption, which limits the application of conventional fluxgate sensors in many areas where compact size and portability are always in demand [4,5]. In the past few years, with the development of information technology and portable electronic equipment, there has been an urgent demand for miniaturized fluxgate sensors in these modern applications [6]. Recently, significant progress has been made in complementary metal–oxide–semiconductor (CMOS)- and microelectromechanical system (MEMS)-based microsensor manufacturing techniques with the rapid development of microelectronics technology [7,8]. Micro-fluxgate sensors fabricated by CMOS and MEMS technology show great potential in many application fields such as parallel robot applications [9], small satellite positioning [10], portable global positioning system (GPS) positioning equipment [11], detection of biomagnetic nanoparticles [12], and global navigation satellite systems [13] because of their small size, light weight, good integration of signal processing circuits, and so on. However, because of the limitation of device dimensions and the saturation magnetizing principle of operation, micro-fluxgate sensors also have several problems, including a low signal-to-noise ratio, relatively poor sensitivity, and a narrow linearity range [14]. Although the signal-to-noise ratio can be compensated for by some additional methods, such as structural optimization or residence time difference technology (RTD) [15], the linearity range and sensitivity, which are severely affected by the core material of the fluxgate sensor [16], are key factors to determining the application performance of the sensor. So far, permalloy is the most traditional soft magnetic material, and it is widely used as the magnetic core material of fluxgate sensors. However, because of its poor high-frequency performance and relatively low saturation induction density, permalloy is far from being satisfactory as the magnetic core material of micro-fluxgate sensors, which are needed in order to meet urgent demand for the development of information technology and portable electronic equipment. In order to facilitate the uninterrupted development of fluxgate sensors toward wider measuring ranges and higher sensitivities, magnetic core materials with better magnetic performance are needed.

To meet this need, amorphous soft magnetic alloys have been developed [17]. As compared to traditional soft magnetic materials, amorphous alloys possess many advantages such as higher permeability, high saturation induction density, and low consumption [17]. Among a variety of amorphous materials, Fe–Co–B amorphous alloys have attracted the attention of researchers in recent years because of their superior soft magnetic properties [18]. As compared to most commercially used Fe-based or Co-based amorphous alloys, Fe–Co–B amorphous materials not only possess the high magnetic induction and high saturation magnetic field strength of Fe-based amorphous alloys [19] but also the low coercivity and low magnetostriction of Co-based amorphous materials [20]. Moreover, because of the excellent high-frequency performance of Fe–Co–B amorphous alloys [21], they are considered a better choice for use as magnetic core materials in high-frequency magnetic sensors. However, by now there is a paucity of studies on the use of amorphous alloys as core materials in micro-fluxgate sensor applications because amorphous soft magnetic alloys are incompatible with the microfabrication process. Studies on Fe–Co–B amorphous alloy-based micro-fluxgate sensors are thus virtually nonexistent.

The goal of the current study was to develop a high-performance micro-fabricated fluxgate sensor associated with the advantages of a Fe–Co–B-based amorphous alloy. Thus, a novel MEMS-micro-fluxgate sensor based on a Fe_58.1_Co_24.9_B_16_Si_1_ amorphous ribbon was designed, fabricated, and tested. First, a simple melt-spinning technique was carried out to obtain a Fe–Co–B-based amorphous ribbon composite of Fe_58.1_Co_24.9_B_16_Si_1_. Then, the obtained amorphous ribbon was annealed at 595 K for 1 h to achieve soft magnetic properties. The prepared ribbon showed excellent soft magnetic behavior with a high saturated magnetic intensity (*B*_s_) of 1.74 T and a coercivity (*H*_c_) of less than 0.2 Oe. Afterward, a micro-fluxgate sensor based on the prepared amorphous ribbon was fabricated via MEMS technology combined with chemical wet etching.

In addition to the properties of core materials, Li et al. [22] found that a higher cross-sectional area of the sensor core can improve the sensitivity of a fluxgate sensor. Furthermore, Jie et al. [23] and Ripka et al. [24] proved that the larger cross-sectional area of the magnetic core not only increases sensitivity but also lowers noise. However, the demagnetization effect and eddy current effect limited our ability to increase the width and thickness of the sensor core. Thus, in the current study, a double-layer core structure was designed in order to increase the cross-sectional area and improve the sensitivity of our sensor. The resulting sensor exhibited a sensitivity of 1985 V/T and a linearity range of ±1.05 mT under optimal excitation conditions. As compared to previously reported similar fluxgate sensors, our sensor showed significantly improved sensitivity and linearity range. Moreover, the sensor performance in terms of offset stability, perming error, and output noise was deeply investigated.

## 2. Materials and Methods

### 2.1. Preparation of the Fe_58.1_Co_24.9_B_16_Si_1_ Amorphous Alloy

Previous studies have shown the influence of the elemental composition on the performance of Fe–Co–B amorphous alloys [18,20,25]. Considering the soft magnetic properties of the alloy, an optimized composition of Fe_58.1_Co_24.9_B_16_Si_1_ was used to fabricate the amorphous materials as follows. Fe_58.1_Co_24.9_B_16_Si_1_ alloy ingots were prepared by a high-frequency induction melting technique. A mixture of high-purity metals (99.9 mass% pure Fe, Co) and metalloids (99.9 mass% Si and 99.5 mass% B) was melted in a pure argon gas atmosphere after evacuation up to 10^−3^ Pa. The ingots were repeatedly smelted five times to ensure the even composition of the alloy. Then, the fabricated Fe_58.1_Co_24.9_B_16_Si_1_ alloy ingots were used to prepare an amorphous ribbon 4–7 mm in width and 10 μm in thickness by a single-roller melt spinner. The melt was overheated to approximately 150 °C above its liquidus temperature and ejected from a quartz crucible through a rectangular orifice by the overpressure of argon (approximately 20 KPa) onto the surface of a smooth cold-rolled copper wheel with a diameter of 550 mm and a circumferential velocity of 50 m/s, which was sufficient to prevent the material from crystallizing.

**Figure 1:**
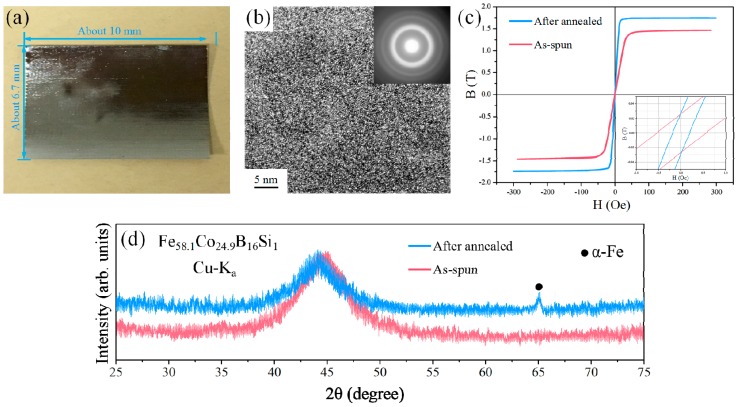
(**a**) Photograph of the fabricated Fe–Co–B ribbon; (**b**) transmission electron microscopy (TEM), high-resolution transmission electron microscopy (HRTEM) image of the Fe–Co–B alloy ribbon; the selected area electron diffraction (SAED) pattern is shown in the inset; (**c**) magnetizing force vs. magnetic flux density (H–B) hysteresis loops for the Fe–Co–B alloy ribbon in the as-spun and annealed (595 K, 1 h) states, the inset shows the partial enlargement for −1~1 Oe; (**d**) XRD pattern of the melt-spun and annealed Fe–Co–B alloy ribbon.

High-resolution transmission electron microscopy (HRTEM) was used to determine the amorphous nature of the alloy. Figure 1a,b shows the photograph and HRTEM image of the as-spun Fe_58.1_Co_24.9_B_16_Si_1_ ribbon. As shown in Figure 1b, a clearly random arrangement of atoms can be noticed. The selected area electron diffraction (SAED) pattern in the inset of Figure 1b shows a classical diffuse diffraction ring without spots. Such features are well known for amorphous materials. In previous works, Li et al. [18] and Han et al. [25] investigated the effect of heat treatment on the soft magnetic properties of Fe–Co–B alloys, and they found that an annealing temperature of 595 K can significantly improve the soft magnetic performance of this material. Based on this, the fabricated amorphous ribbon was annealed at 595 K for 1 h to achieve the soft magnetic properties. The magnetizing force versus magnetic flux density (H–B) measurements were carried out by using a vibrating sample magnetometer (VSM) to determine the soft magnetic behavior of our ribbon. As shown in Figure 1c, the fabricated ribbon clearly exhibited an increased magnetic intensity (*B*_s_) after annealing. Generally speaking, the saturation magnetic intensity of materials usually remains the same if the chemical composition does not change. To further explore this problem, X-ray diffraction (XRD) with Cu K_a_ radiation was utilized to determine the inner structure of the ribbon before and after annealing. As can be observed from Figure 1d, the presence of a broad halo without sharp peaks suggests the amorphous nature of this ribbon before heat treatment. However, an α-Fe crystallization trend can clearly be found in the ribbon after annealing. In a previous study, Makino et al. [26] indicated that the α-Fe nano-crystal may improve the soft magnetic properties of soft magnetic material owing to the high saturation intensity of α-Fe. Consequently, the α-Fe crystallization in our material after annealing may be the main reason contributing to the increasing saturation magnetic intensity of the ribbon. After annealing, the prepared ribbon shows a coercivity (*H*_c_) of less than 0.2 Oe and a high saturated magnetic intensity (*B*_s_) of 1.74 T. Such a *B*_s_ value is much higher than that of traditional commercially used permalloy (0.6–1 T) or the commercialized Co-based (0.77–1.1 T) and Fe-based (1.5 T) amorphous ribbon. However, in the most recently reported works of Wang et al., an ultrahigh *B*_s_ value of 1.85 T [18] and 1.92 T [27] were achieved in a Fe–Co-based amorphous alloy with similar composition as ours by magnetic field heat treatment. This indicates that the soft magnetic properties of our materials can be further improved by more optimized annealing process.

### 2.2. Fabrication of the Micro-Fluxgate Sensor

**Figure 2:**
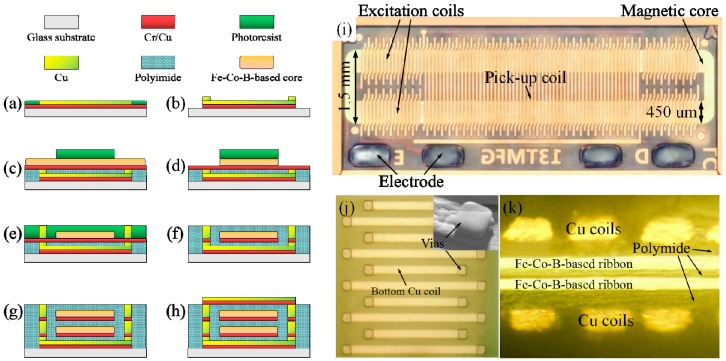
(**a**–**h**) The detailed fabrication process of the micro-fluxgate sensor; (**i**) the fabricated micro-fluxgate sensor; (**j**) images of the bottom coil and vias; (**k**) the cross-sectional image of the sensor.

The fabrication of the micro-fluxgate sensor was performed on a circular glass wafer. Figure 2a–h shows the MEMS-based fabrication process of the microsensor as follows: (a) A Cr–Cu seed layer was sputtered onto the glass substrate as conductive layer for electroplating, and a positive photoresist was spun on the seed layer, which was then patterned by ultraviolet lithography with a template of the bottom coil. Then, a 20-μm-thick Cu film was electroplated in the photoresist mold to act as the bottom coil. (b) After the bottom coil was completed, the positive photoresist was spun and patterned with a template of vias. A vertical Cu cylinder was electroplated in the photoresist mold to act as the Cu vias to connect the top coil. Then, the seed layer was removed by reactive ion etching after the photoresist was eliminated with acetone. (c) Polyimide was spun onto the wafer and baked at 250 °C in vacuum for 2 h for solidification; here, polyimide was used for electrical insulation in order to isolate the magnetic core from the bottom Cu coil. Then, the polyimide was etched by reactive ion etching to expose the Cu vias. Then, another Cr–Cu seed layer was deposited onto the surface and a Fe–Co–B-based magnetic core (Fe_58.1_Co_24.9_B_16_Si_1_) with a thickness of 10 μm was attached onto the wafer by epoxy AB glue. (d) Because of the present processing of MEMS technology, how to fabricate an amorphous core with a particular size and shape is often a difficult problem to resolve. In this work, chemical wet etching was adopted in the fabrication of the Fe–Co–B-based amorphous magnetic core. In order to pattern the magnetic core, a photoresist model was made on the surface of the ribbon and patterned with the shape of the sensor core. Then, chemical wet etching (etching solution: 1HNO_3_:2HCl:4H_2_O_2_:8H_2_O, in volume ratio) was used to remove the excess ribbon uncovered with the positive photoresist, and the Fe–Co–B amorphous ribbon was etched into a long rectangular shape for use as the magnetic-sensitive core of our sensor. The epoxy dispergator was then utilized to remove the residual glue. (e) After eliminating the photoresist, a new positive photoresist was spun and patterned with the template of vias. The Cu vias were then electroplated to reach a height over the magnetic core. (f) Afterwards, the Cr–Cu seed layer was removed by reactive ion etching after eliminating the photoresist, and polyimide was spun on the wafer again and baked at 250 °C in vacuum for 2 h to isolate the sensor core from the top coils. Then, the polyimide was etched by reactive ion etching to expose the Cu vias. (g) A second layer of magnetic core and vertical vias was fabricated using the same sequence of lithography, electroplating, and polyimide discussed above in Figure 2c–f. (h) After fabrication of the sensor core and coating the Cr–Cu seed layer on the wafer again, the positive photoresist was spun onto the seed layer and patterned with the template of the top Cu coil. A Cu film with a thickness of 20 μm was then electroplated in the photoresist mold to form the top Cu coil. Finally, the whole sensor was obtained after removing the photoresist and seed layer. Photographs of the fabricated sensor are shown in Figure 2i–k. As shown in Figure 2i,k, the fabricated microsensor contained a long rectangular magnetic core (7.3 mm × 1.5 mm, 450 μm in width for long side) as the sensitive element of the sensor, and a double-layer sensor core made of an Fe–Co–B amorphous ribbon was used to increase the cross-sectional area of the magnetic core, which increased the sensitivity of our sensor [22,23,24]. Three-dimensional solenoid Cu coils (one pick-up coil and four excitation coils) were applied to control the magnetic-sensitive elements of the sensor. The excitation coils (16 turns for each coil, line width of 60 μm) were employed to drive the sensor core periodically into the magnetic saturation state in response to the excitation current generated in the excitation coils. The pick-up coil (59 turns, line width of 60 μm) was utilized to pick up the permeability variation of the sensor core when an external magnetic field was tested. The pick-up coil was located parallel to and between the four excitation coils. The dimensions of the entire sensor measured with an outer size of 7.3 mm in length and 2.3 mm in width (not including the electrode) or 7.3 × 2.7 mm^2^ (including the electrode).

### 2.3. Testing System of the Micro-Fluxgate Sensor

**Figure 3:**
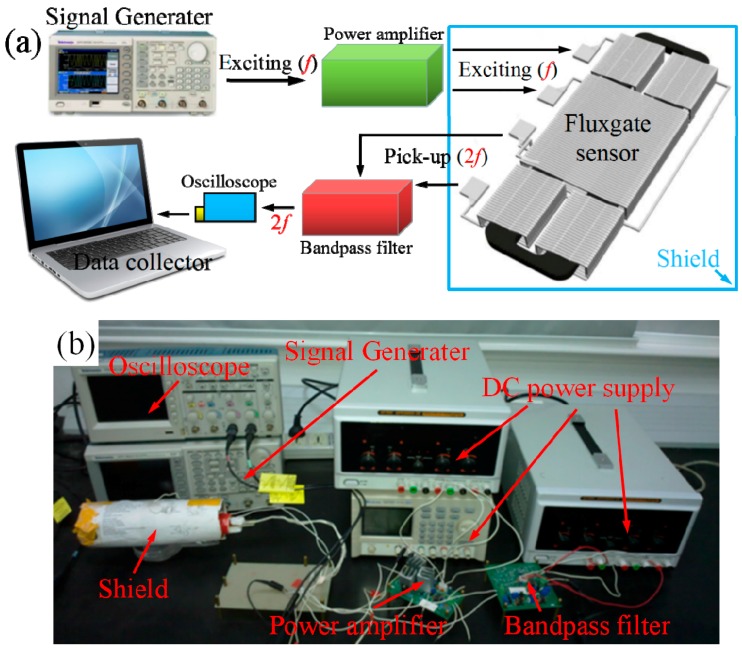
(**a**) Schematic illustration of test system circuit; (**b**) Photograph of the test system.

Generally, a fluxgate sensor measuring system is based on the second harmonic principle that consists of excitation and sensing circuits. The excitation circuits must ensure the magnetic core is working in a deep saturation state, which results in a noticeable variation of permeability of the core when an external magnetic field is added. The sensing circuits should be able to pick up the second harmonic signal effectively from the output of the pick-up coil. In this work, we established a measuring system that included a signal generator, a power amplifier, a biquadratic bandpass filter, and an oscilloscope. A block diagram of the measuring system is shown in Figure 3a.

The signal generator (Tektronix AFG 3022, Tektronix, Beaverton, OR, USA) was set to provide a sine wave signal. Because the power of the signal provided by the generator was too small to drive the fluxgate sensor, a power amplifier printed circuit board (PCB) power amplifier circuit, (see Figure S1 in detail) was needed to ensure a powerful enough excitation signal. A biquadratic bandpass filter (Mitron LPFD-3040+, Mitron Interlink, Inc., San Chung, Taiwan) was used to pick up the second harmonic signal from the output signal of the pick-up coil. The measuring results were read with the oscilloscope (Tektronix TDS2014B, Tektronix, Beaverton, OR, USA). In order to guarantee the accuracy of the test results, a cylindrical magnetic shield (10 layers of FeNi thin films) was used to protect of our sensor from the environmental magnetic field. Figure 3b shows a photograph of the test system.

## 3. Results and Discussion

### 3.1. Sensitivity and Linearity

**Figure 4:**
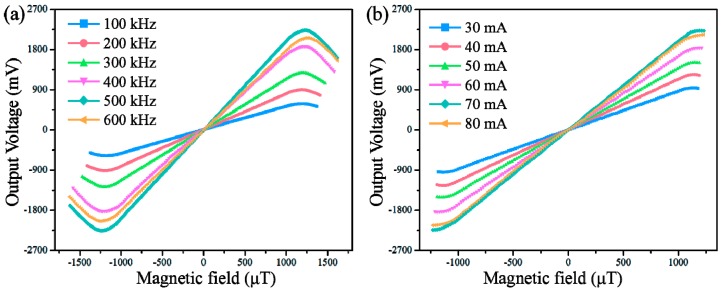
(**a**) The sensor response for different levels of excitation frequency at an excitation current of 70 mA; (**b**) The sensitivity of the sensor as a function of the magnitude of the excitation current.

The sensor was first tested with several excitation frequency values and excitation current amplitudes in order to find the optimum operating conditions. The sensitivity measurement was implemented by applying an external direct current (DC) magnetic field to the sensor by a solenoid coil (Tian Heng Control Technology Co., Ltd., Tianjin, China, amplitude of ±1.92 mT), and the outside of the coil was covered with 10 layers of FeNi thin films to form a magnetic shield. As shown in Figure 4a, we clearly observed that a higher driving frequency resulted in greater sensitivity of the sensor. However, when the frequency value exceeded 500 kHz, a clear decrease in sensitivity was found in our sensor response. According to the operation function of the second harmonic principle, the sensitivity empirical formula for a long-rectangular-core fluxgate sensor can be simplified as follows [28]:

(1) $$S_{\max}=8fNA\mu_{a}$$

where *S*_max_ is the maximum sensitivity of the fluxgate sensor, *f* is the excitation frequency, *N* is the number of turns of the pick-up coil, *A* is the cross-sectional area of the magnetic core, and *μ*_a_ is the effective longitudinal apparent permeability for the rectangular prism core, which is always proportional to the saturated magnetic induction (*B*_s_) of the core materials.

From Equation (1), it is evident that the sensitivity of the fluxgate sensor can be linearly enhanced by increasing the drive frequency, as shown in Figure 4a. However, at an overlarge excitation frequency (over 500 kHz in this work), increasing eddy current losses and the demagnetization effect at high frequency may significantly decrease the effective permeability (*μ*_a_) of the sensor core, resulting in a decrease in sensor response [7]. Based on this, an excitation frequency of 500 kHz, which was proven to be an optimal condition for the sensitivity of the fabricated sensor, and the same excitation conditions were used in the subsequent experiments.

The changes of the sensitivity with different excitation current amplitudes were also investigated and the results are shown in Figure 4b. It is apparent that the sensitivity of our sensor increased with increasing excitation current amplitude and then remained nearly the same after a current amplitude over 70 mA. This phenomenon is mainly attributed to the influence of the excitation current on the saturation extent of the sensor core: a larger current amplitude always means a deeper saturation state of the magnetic core of the sensor, resulting in higher sensitivity in the output response. However, for the excitation current of 70 mA, the sensor core may have become fully saturated, and therefore no significant difference in sensor sensitivity was observed when the current amplitude exceeded 70 mA. Thus, the optimum excitation current of 70 mA rms (root mean square) was used for subsequent experiments unless otherwise noted. A maximum sensitivity of 1985 V/T was achieved under the excitation current amplitude of 70 mA at an excitation frequency of 500 kHz.

**Figure 5:**
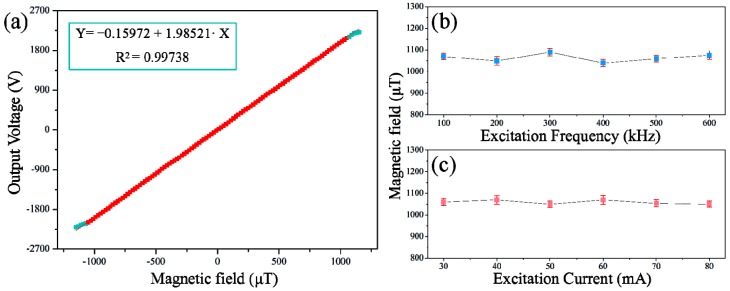
(**a**) Linearity analysis of sensor response under the excitation current amplitude of 70 mA at an excitation frequency of 500 kHz; (**b**) The linear range of the sensor with different excitation frequencies; (**c**) The linear range of the sensor with different excitation current amplitudes.

Figure 5a indicates the relation of outputs to the external magnetic field under optimum excitation conditions. A straightforward linear relationship between the magnetic field values and the output voltage of the sensor is observed. The linear regression equation is expressed as Y = −0.15972 + 1.98521X with a correlation coefficient of 0.99738 in the linear range of approximately ±1.05 mT. The effect of excitation current magnitude and frequency on the linearity range of our sensor was also studied and the results are shown in Figure 5b,c, respectively. The results demonstrate the independence of the linear range on the excitation current amplitude and excitation frequency. Similar results were found in a previous study by Zorlu et al. [14], where the linear operation range of a fluxgate sensor had no relation with the excitation conditions; it was, however, directly affected by magnetic core materials due to the independence of the excitation and detection mechanisms.

### 3.2. Offset Stability

**Figure 6:**
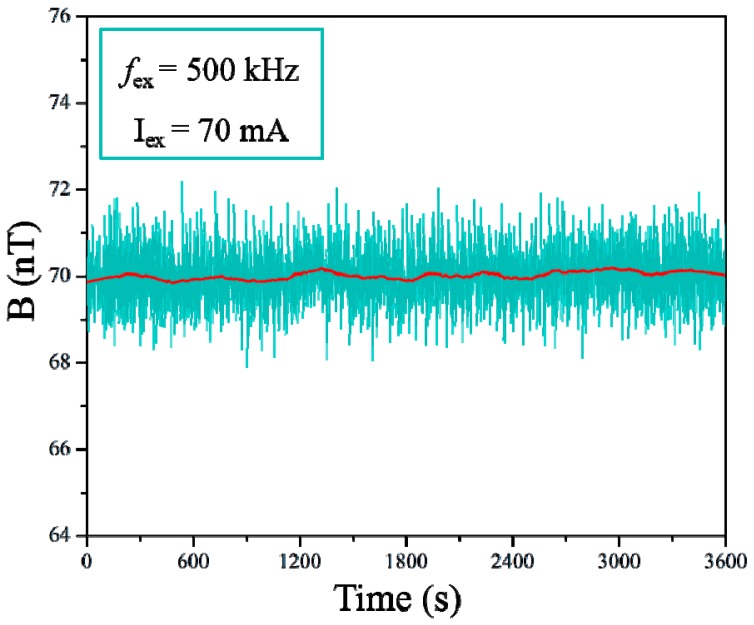
Offset drift of the sensor over 1 h.

The long-term offset stability of the current sensor was measured by observing the outputs over 12 h. The sensor was placed in a shielded environment (cylindrical magnetic shield made of 10 layers of FeNi thin films) with zero applied field. Figure 6 shows the offset of this sensor for a 500-kHz excitation frequency and a 70-mA excitation current. Because the magnitudes of the offset changes were similar, only a 1-h excerpt is shown. After warming up, the offset changes showed a bandwidth of approximately 4.3 nT. In previous studies, Kubik et al. [2] reported a printed circuit board (PCB)-based micro-fluxgate sensor with an offset stability of 21 nT bandwidth and Trigona et al. [3] reported an RTD technology-based microwire-fluxgate sensor with an improved offset stability of up to approximately 6 nT bandwidth. As compared to similar microsensors reported, the current sensor exhibited superior stability performance.

### 3.3. Perming Error

**Figure 7:**
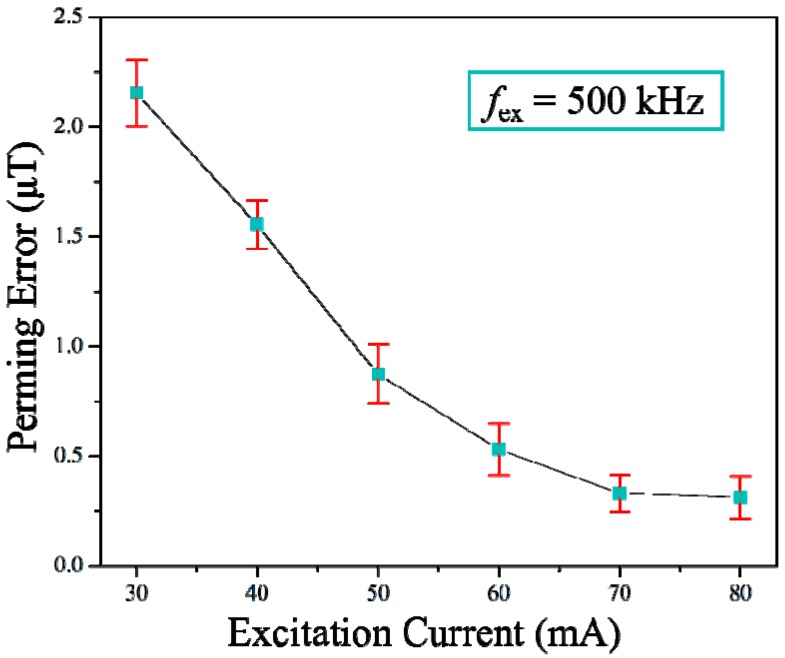
The perming error of the sensor as a function of excitation current magnitude.

The perming effect is a parasitic response of ferromagnetic materials to the application of strong magnetic field pulses. It appears as an offset change (drift of zero-field value) of the sensor after applying such pulses. The perming error was investigated by applying a magnetic shock to the sensor by using a current-controlled Helmholtz coil with an amplitude of 20 mT. We then calculated the offset changes from the outputs. Figure 7 shows the variation of the perming error of the sensor with the different excitation currents. It is apparent that the perming decreased with increasing excitation current due to the deeper saturation state of the magnetic core under the larger driven excitation current amplitude. With a 70-mA excitation current at a frequency of 500 kHz, the sensor showed a perming error below 0.4 μT.

### 3.4. Noise

**Figure 8:**
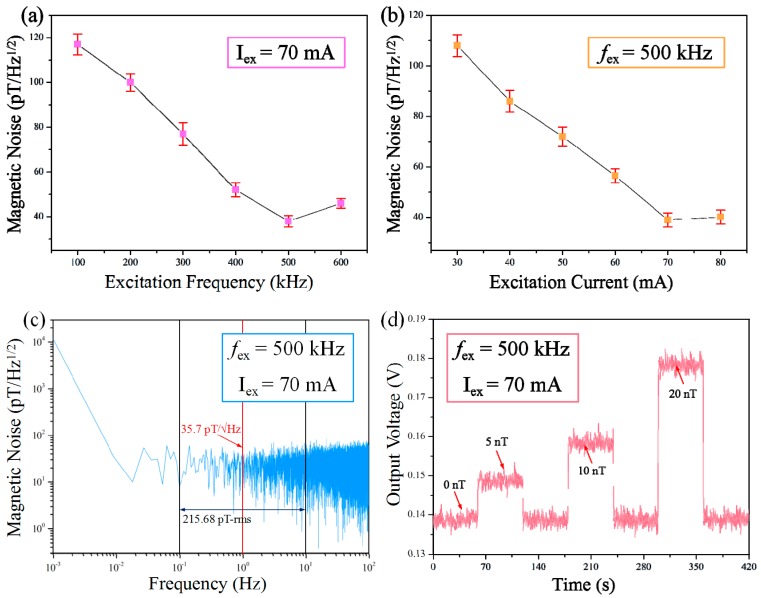
(**a**) Effect of excitation frequency on the magnetic noise of the sensor; (**b**) Effect of excitation current value on the magnetic noise of the sensor; (**c**) The magnetic noise spectrum of the sensor up to 10 Hz for 70 mA peak excitation current at 500 kHz frequency; (**d**) Time-domain response of the sensor for four different external field values.

In addition to the sensitivity and linearity, the signal-to-noise ratio is also a key factor in assessing the performance of sensors in practical applications. The magnetic noise tests of our sensor were first carried out under different excitation frequencies and current amplitude conditions at 1 Hz in pT/Hz^1/2^. As can be seen in Figure 8a, there exists a minimum noise value at 500 kHz when the excitation frequency is considered. This can be attributed to the change in the alternating current (AC) magnetic properties such as eddy current losses and demagnetization effect in the ferromagnetic layer toward variable driven frequencies. However, the noise level of our sensor clearly decreased with increasing excitation current amplitude because the melt-spinning ferromagnetic layer showed improved saturation with higher excitation current values (Figure 8b). Figure 8c presents the equivalent magnetic noise spectrum of the sensor for 70 mA peak excitation at 500 kHz frequency in a shielded environment. The measured magnetic noise is about 36 pT/Hz^1/2^ at 1 Hz and the noise rms level is 215.68 pT within 0.1–10 Hz. Figure 8d shows the time-domain noise information of the sensor for four different external magnetic field values (0, 5 nT, 10 nT, 20 nT) under an excitation current amplitude of 70 mA and a frequency of 500 kHz, which corresponds to the minimum noise conditions according to Figure 8a–c. As shown in Figure 8d, evident stage differences in the sensor response were observed when the micro-fluxgate sensor was exposed to different external magnetic fields. The output curve of each magnetic field can be clearly distinguished, and a minimum external field of 5 nT can be clearly detected by our sensor, which indicates the superior resolution of our sensor. Although the resolution level is still fairly large as compared to those of commercially used conventional fluxgate sensors, it is still very good for a magnetic sensor with microstructure. To the best of our knowledge, a compact micro-fluxgate sensor with such a high-resolution capability has not been reported anywhere else.

Moreover, a comparison was made with respect to the performance of recently reported magnetic sensors and some commercialized sensors (as shown in Table 1). Compared with the sensors in most other studies, the results indicated that the micro-fluxgate sensor in this work possessed a wide linearity range and a relatively high sensitivity. Although the reported magnetoelectric composite (MC)-based sensor [31] or the commercial giant magnetoimpedance (GMI) sensor (Type DH) by AICHI Micro Intelligent Co., Ltd., Tōkai, Japan [33] and commercial fluxgate sensor (Type uMag-01/02) by MEDA Co., Ltd., Tianjin, China [36] show higher sensitivity or resolution than ours, our sensor presents a great advantage over the linearity range performance. Actually, even when compared with certain commercial sensors [33,34,35,36,37,38], our sensor also exhibits an excellent comprehensive performance, especially in the linear range property. In addition, when compared with the commercialized system, the test system in our proposed work for determining our sensor is relatively simple and an open-loop circuit. In a previous study, Snoeij et al. [39] and Yang et al. [40] indicated that adding a feedback control unit into test system forms a closed-loop circuit, which may clearly improve the detection performance of a fluxgate sensor. This indicates that our sensor performance still had the potential to be further improved by operation circuit-loop optimization.

## 4. Conclusions

A novel fluxgate sensor with a bilayer Fe–Co–B-based amorphous ribbon core was designed, fabricated, and tested in the current study. A simple melt-spinning technique was carried out to obtain a Fe–Co–B-based amorphous ribbon composite of Fe_58.1_Co_24.9_B_16_Si_1_. Then, the obtained as-spun ribbon was annealed at 595 K for 1 h to achieve soft magnetic properties. The prepared material showed excellent soft magnetic performance, with a high saturated magnetic intensity of 1.74 T and a coercivity of less than 0.2 Oe. Afterward, a micro-fluxgate sensor based on the prepared amorphous ribbon was fabricated via MEMS technology combined with chemical wet etching. The resulting sensor exhibited a sensitivity of 1985 V/T, a wide linearity range of ±1.05 mT, and a perming error below 0.4 μT with a 70-mA excitation current and a 500-kHz frequency. The minimum magnetic field noise was about 36 pT/Hz^1/2^ at 1 Hz under the same excitation conditions, and a superior resolution of 5 nT was also achieved in the fabricated sensor. To the best of our knowledge, a compact micro-fluxgate sensor with such a high-resolution capability has not been reported anywhere else. When compared to similar magnetic sensors previously reported [7,31,32,33,34], our sensor not only exhibited a relatively high sensitivity but also provided a wide measuring linearity range. Moreover, because the current fluxgate sensor can be easily fabricated via the MEMS technique and is compatible with lab-on-chip technology, it can be easily integrated into an electronic microchip for modern information technology and portable electronic equipment applications. In summary, the microsensor presented here with such improved characteristics may considerably enhance the development of micro-fluxgate sensors and is promising for more application fields.

## Figures and Tables

**Table 1:** Comparison of recently reported magnetic sensors.

Materials, Methods	Linearity Ranges	Sensitivity (V/T)	Size	Resolution	Noise Level	Operating Current	Reference
Permalloy-based MEMS-micro-fluxgate sensor	±300 μT	327	3 × 4 mm^2^	--	--	150 mA	[29]
Co-based amorphous ribbon fluxgate sensor	±1 mT	593	3 × 6.5 cm^2^	--	790 pT/Hz^1/2^	600 mA	[7]
Co-based amorphous ribbon giant magnetoimpedance (GMI) sensor	~±1 μT	~1800	1 × 9 mm^2^	--	17 pT/Hz^1/2^	20 mA	[30]
Magnetoelectric composite-based sensor	~1 nT–1 μT	3800	4 × 4 mm^2^	--	27 pT/Hz^1/2^	--	[31]
Hall sensor based on bilayer graphene	±8 mT	32	0.7 × 2.1 mm^2^	118 μT	--	1.2 mA	[32]
Commercialized GMI sensor (Type DH) by AICHI micro intelligent Co., Ltd., Tōkai, Japan	±40 μT	10^6^	35 × 11 mm^2^	1 nT	30 pT/Hz^1/2^	15 mA	[33]
Commercialized HMR sensor (Type 3300) by HoneyWell Co., Ltd., Seoul, Korea	±200 μT	--	82 × 38 mm^2^	10 nT	--	35 mA	[34]
Commercialized HMR sensor (Type 2300-D21-485) by HoneyWell Co., Ltd., Seoul, Korea	±40 μT	--	25 × 30 mm^2^	6.7 nT	--	27 mA	[35]
Commercialized Fluxgate sensor (Type uMag-01/02) by MEDA Co., Ltd., Tianjin, China	±2 μT~±200 μT	--	12 × 27 mm^2^	1 nT	--	--	[36]
Commercialized Fluxgate sensor (Type Mag619) by Bartington Co., Ltd., Witney, UK	±60 μT	--	25 × 20 mm^2^	Several nT	≤50 pT/Hz^1/2^	38 mA	[37]
Commercialized TMR sensor (Type TMR9003) by Dowaytech Co., Ltd., San Jose, CA, USA	±1.5 mT	300	6 × 5 mm^2^	--	750 pT/Hz^1/2^	20 μA	[38]
This work	±1.05 mT	1985	2.7 × 7.3 mm^2^	5 nT	36 pT/Hz^1/2^	70 mA	Current study

## Supplementary Materials

The Figure S1 is available online at www.mdpi.com/2072-666X/8/12/352/s1.
