# Supplementary material for: Wide Linearity Range and Highly Sensitive MEMS-Based Micro-Fluxgate Sensor with Double-Layer Magnetic Core Made of Fe–Co–B Amorphous Alloy
Source: Micromachines (Basel). 2017 Nov 30;8(12):352. doi: 10.3390/mi8120352 (PMC6187929; doi:10.3390/mi8120352)
Supplement: Supplementary file 1 [file micromachines-08-00352-s001.pdf]

Detailed information about the power amplifier chip

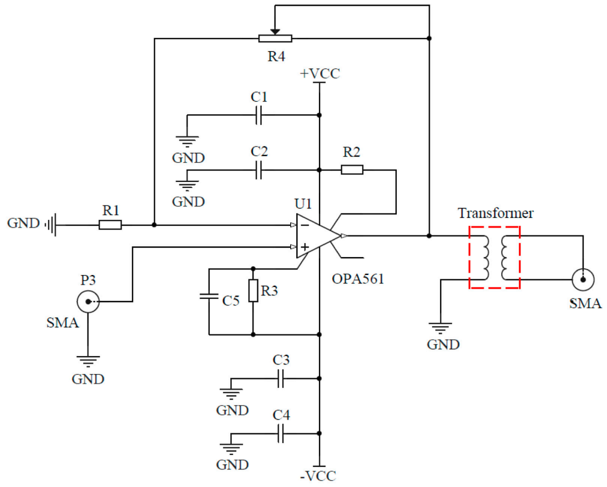

Figure S1. Diagram of the power amplifier

We designed and welded the power amplifier chip on a PCB board. The circuit-loop of this PCB power amplifier chip is shown in Figure S1. An OPA561 chip (Texas Instruments Co., Ltd.) is used as the power amplifier chip (maximum output: 1.2 A, maximum output voltage: 12 V_{pp}, full power bandwidth of 12 MHz), and an additional series transformer DA101C (turns ratio = 1:1, working frequency < 3 MHz, minimum return loss of 46.8 dB, Arrow Electronics Co., Ltd., Centennial, CO, USA) was used to block the DC component of the excitation current to ensure the equality of positive and negative signal.
